# Supplementary material for: Subspecies Niche Specialization in the Oral Microbiome Is Associated with Nasopharyngeal Carcinoma Risk
Source: mSystems. 2020 Jul 7;5(4):e00065-20. doi: 10.1128/mSystems.00065-20 (PMC7343305; doi:10.1128/mSystems.00065-20)
Supplement: TABLE S1 [file mSystems.00065-20-st001.docx]

|  | |  | **Cases**  **(n=499)** | | **Controls**  **(n=495)** | | **p-value^§^** |
| --- | --- | --- | --- | --- | --- | --- | --- |
| Mean age in years | | | 48.4 | (10.5) | 49.5 | (10.4) | 0.09**^†^** |
| Number of males (%) | | | 356 | (71.3%) | 354 | (71.5%) | 1.99 |
| Educational Attainment | | |  |  |  |  | 6x10^-8^*** |
|  | Less than 6 years | | 208 | (41.7%) | 126 | (25.5%) |  |
|  | 7 – 9 years | | 175 | (35.1%) | 192 | (38.8%) |  |
|  | 10 – 12 years | | 94 | (18.8%) | 119 | (24.0%) |  |
|  | More than 12 years | | 18 | ( 3.6%) | 47 | ( 9.5%) |  |
| Body Mass Index (10 years ago) | | |  |  |  |  | 0.18 |
|  | Underweight | | 51 | (10.2%) | 54 | (10.9%) |  |
|  | Normal | | 370 | (74.1%) | 348 | (70.3%) |  |
|  | Overweight | | 72 | (14.4%) | 78 | (15.8%) |  |
|  | Obese | | 6 | ( 1.2%) | 15 | ( 3.0%) |  |
| Home Community | | |  |  |  |  | 0.0009*** |
|  | Cangwu | | 125 | (25.1%) | 89 | (18.0%) |  |
|  | Cenxi | | 168 | (33.7%) | 160 | (32.3%) |  |
|  | Tengxian | | 97 | (19.4%) | 146 | (29.5%) |  |
|  | Wuzhou | | 109 | (21.8%) | 100 | (20.2%) |  |
| Tobacco Use | | |  |  |  |  | 0.02* |
|  | Never | | 238 | (47.7%) | 273 | (55.2%) |  |
|  | Current | | 261 | (52.3%) | 222 | (44.8%) |  |
| History of Alcohol Use | | |  |  |  |  | 0.34 |
|  | Never | | 339 | (67.9%) | 357 | (72.1%) |  |
|  | Former | | 20 | ( 4.0%) | 15 | ( 3.0%) |  |
|  | Current | | 139 | (27.9%) | 123 | (24.8%) |  |
| Tea Consumption | | | 145 | (29.1%) | 155 | (31.3%) | 0.48 |
| Frequency of Salted Fish Consumption in adulthood | | | | |  |  | 0.77 |
|  | Never | | 193 | (38.7%) | 197 | (39.8%) |  |
|  | Yearly | | 207 | (41.5%) | 202 | (40.8%) |  |
|  | Monthly | | 95 | (19.0%) | 85 | (17.2%) |  |
| Frequency of Salted Fish Consumption in childhood | | | | |  |  | 0.60 |
|  | Never | | 143 | (28.7%) | 146 | (29.5%) |  |
|  | Yearly | | 210 | (42.1%) | 213 | (43.0%) |  |
|  | Monthly | | 142 | (28.5%) | 125 | (25.3%) |  |
| Serum EBV Status | | |  |  |  |  | 1x10^-15^*** |
|  | Negative | | 16 | ( 3.2%) | 346 | (69.9%) |  |
|  | Positive | | 416 | (83.4%) | 110 | (22.2%) |  |
|  | Unknown | | 67 | (13.4%) | 39 | ( 7.9%) |  |
| Family History of NPC | | |  |  |  |  | 6x10^-5^*** |
|  | No | | 432 | (86.6%) | 473 | (95.6%) |  |
|  | Yes | | 54 | (10.8%) | 20 | ( 4.0%) |  |
|  | Missing or Unknown | | 13 | ( 2.6%) | 2 | ( 0.4%) |  |
| History of Rhinitis | | | 18 | ( 3.6%) | 22 | ( 4.4%) | 0.61 |
| History of Pharyngitis | | | 20 | ( 4.0%) | 19 | ( 3.8%) | 0.98 |
| Number of Missing or Repaired Teeth | | | |  |  |  | 0.30 |
|  | 0 | | 217 | (43.5%) | 202 | (40.8%) |  |
|  | 1 | | 63 | (12.6%) | 57 | (11.5%) |  |
|  | 2 | | 53 | (10.6%) | 66 | (13.3%) |  |
|  | 3-5 | | 83 | (16.6%) | 100 | (20.2%) |  |
|  | 6+ | | 83 | (16.6%) | 70 | (14.1%) |  |
| Tooth Brushing Frequency | | |  |  |  |  | 6x10^-11^*** |
|  | <=1/day | | 325 | (65.1%) | 219 | (44.2%) |  |
|  | >=2/day | | 174 | (34.9%) | 276 | (55.8%) |  |

^†^p-value Welch’s t-test

^§^p-value Chi-square test

* p < 0.05; ** p < 0.01; *** p < 0.001
