# Supplementary figures and images for: Subspecies Niche Specialization in the Oral Microbiome Is Associated with Nasopharyngeal Carcinoma Risk
Source: mSystems. 2020 Jul 7;5(4):e00065-20. doi: 10.1128/mSystems.00065-20 (PMC7343305; doi:10.1128/mSystems.00065-20)

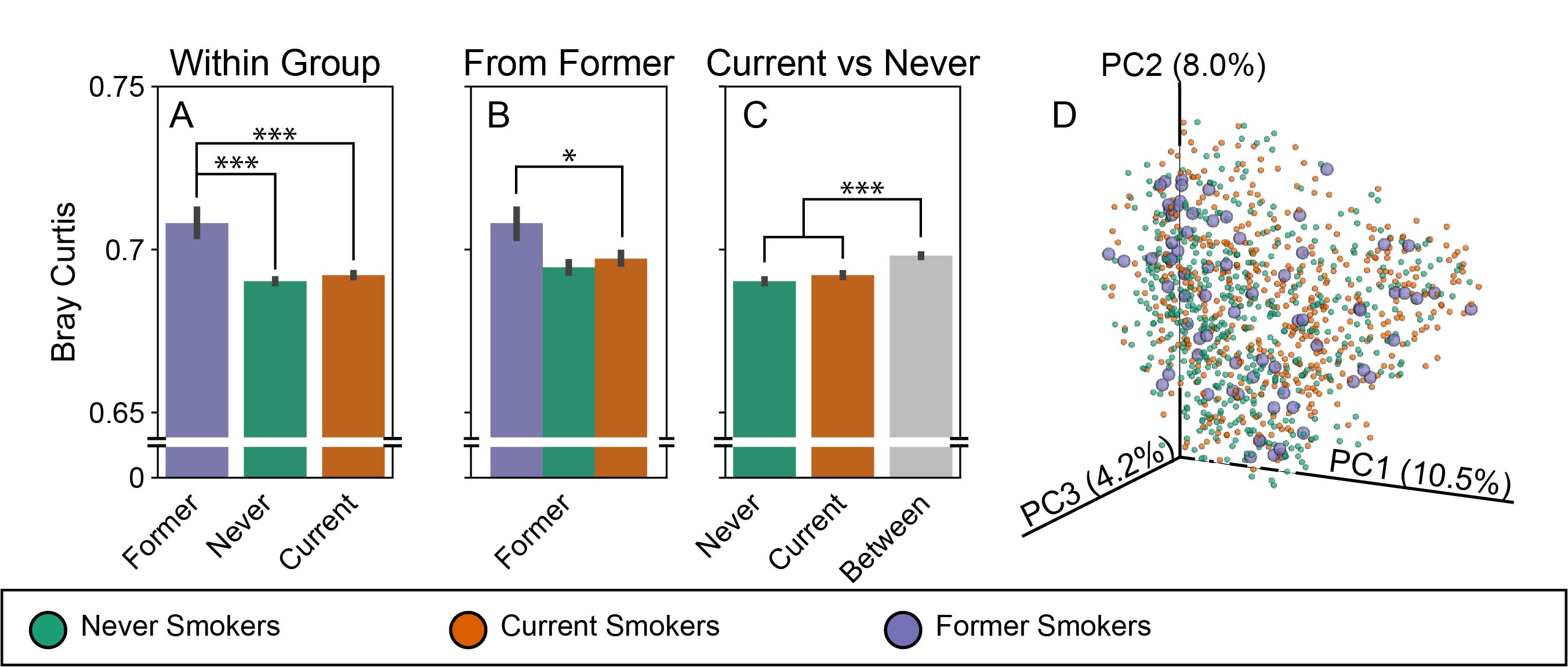

Supplement: FIG S2 [file mSystems.00065-20-sf002.jpg]

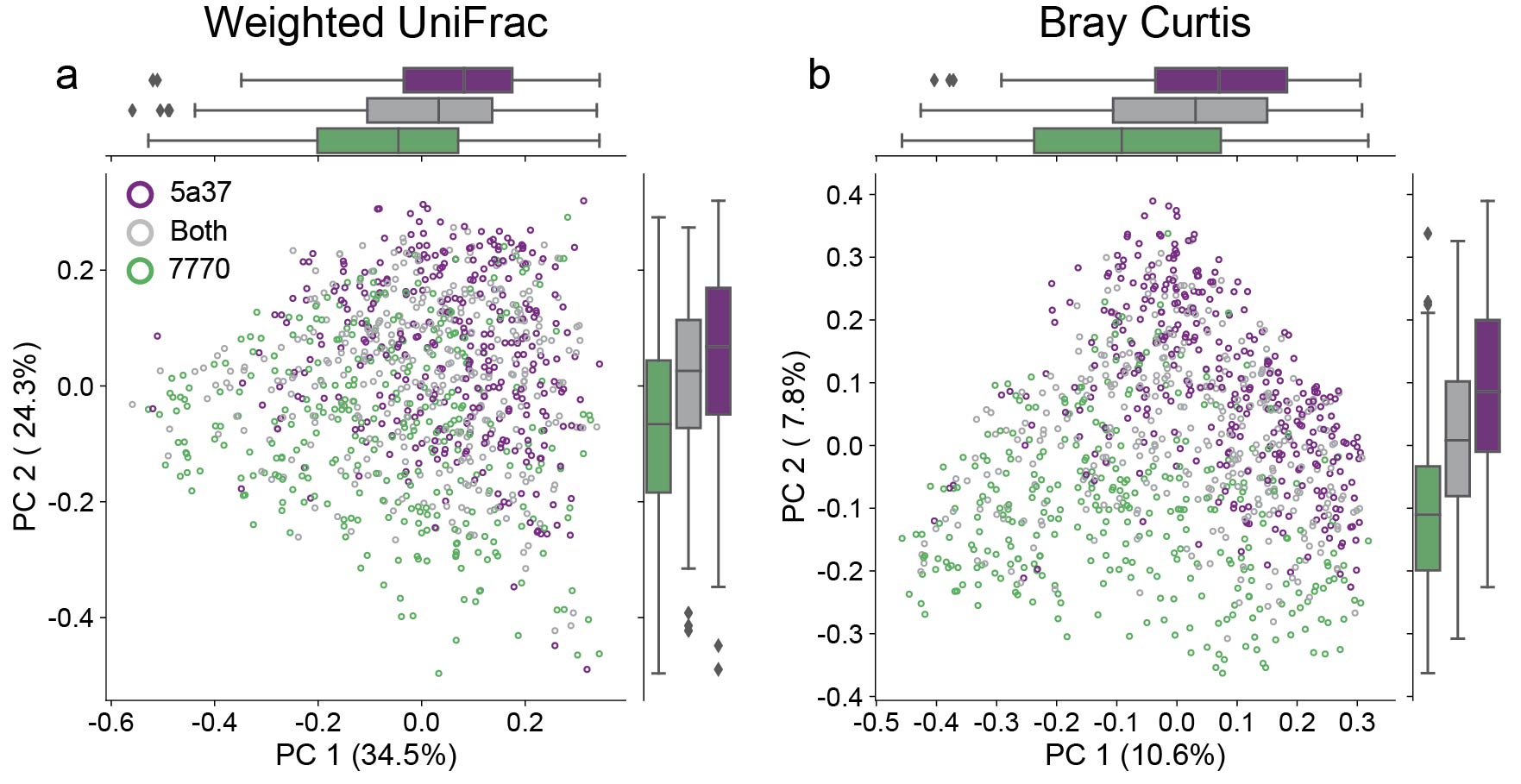

Supplement: FIG S4 [file mSystems.00065-20-sf004.jpg]
