# Supplementary material for: Subspecies Niche Specialization in the Oral Microbiome Is Associated with Nasopharyngeal Carcinoma Risk
Source: mSystems. 2020 Jul 7;5(4):e00065-20. doi: 10.1128/mSystems.00065-20 (PMC7343305; doi:10.1128/mSystems.00065-20)
Supplement: TABLE S2 [file mSystems.00065-20-st002.docx]

|  | **Phylogenetic Diversity** | | **Observed ASVs** | | **Shannon Diversity** | |
| --- | --- | --- | --- | --- | --- | --- |
|  | statistic | p-value | statistic | p-value | statistic | p-value |
| NPC status | 118.7 | < 1x10^-12^* | 172.6 | < 1x10^-12^* | 46.3 | 1x10^-11^* |
| Residential Community | 11.1 | 0.01* | 13.3 | 0.004* | 16.5 | 0.001* |
| Tooth Brushing Frequency | 5.4 | 0.02* | 10.9 | 0.001* | 3.1 | 0.08 |
| History of Pharyngitis | 7.3 | 0.007* | 7.2 | 0.007* | 0.3 | 0.60 |
| Tea Consumption | 1.5 | 0.22 | 0.7 | 0.40 | 5.7 | 0.02* |
| Smoking Status | 0.7 | 0.42 | 0.3 | 0.58 | 4.5 | 0.04* |
| History of Rhinitis | 1.6 | 0.20 | 1.5 | 0.22 | 4.3 | 0.04* |
| Educational Attainment | 4.9 | 0.30 | 5.1 | 0.28 | 7.4 | 0.12 |
| BMI 10 years ago | 3.0 | 0.39 | 4.5 | 0.21 | 1.9 | 0.59 |
| Childhood Salted Fish Consumption | 1.9 | 0.38 | 1.2 | 0.54 | 0.2 | 0.90 |
| Family History | 0.2 | 0.67 | 0.1 | 0.78 | 0.6 | 0.45 |
| Missing or Repaired Teeth | 2.5 | 0.64 | 3.7 | 0.45 | 3.7 | 0.45 |
| Adult Salted Fish Consumption | 1.5 | 0.47 | 1.3 | 0.58 | 0.3 | 0.84 |
| History of Alcohol Use | 0.2 | 0.66 | 0.3 | 0.60 | 0.0 | 0.96 |

*Significant at α < 0.05
