# Supplementary material for: Subspecies Niche Specialization in the Oral Microbiome Is Associated with Nasopharyngeal Carcinoma Risk
Source: mSystems. 2020 Jul 7;5(4):e00065-20. doi: 10.1128/mSystems.00065-20 (PMC7343305; doi:10.1128/mSystems.00065-20)
Supplement: TABLE S5 [file mSystems.00065-20-st005.docx]

| **Cluster** | **Node** | **Clone Name** | **HOMD-ID** | **Status** | **Identity (%)** |
| --- | --- | --- | --- | --- | --- |
| 1 | cPre-1412 | Alloprevotella sp. HMT 308 | HMT-308 | Phylotype | 99.8 |
|  | Gran-7770 | Granulicatella adiacens | HMT-534 | Named | 100. |
|  | Prev-4b21 | Prevotella histicola | HMT-298 | Named | 99.8 |
|  | Prev-b7f2 | Prevotella melaninogenica | HMT-469 | Named | 100. |
|  | Prev-cefa | Prevotella salivae | HMT-307 | Named | 100. |
|  | Bull-4893 | Solobacterium moorei | HMT-678 | Named | 99.5 |
|  | Stre-900d | Streptococcus parasanguinis clade 411 | HMT-411 | Named | 100. |
|  | Stre-b566 | Streptococcus salivarius | HMT-755 | Named | 100. |
|  | Veil-8f88 | Veillonella atypica | HMT-524 | Named | 100. |
|  | Veil-98e7 | Veillonella atypica | HMT-524 | Named | 99.8 |
|  | Veil-675d | Veillonella dispar | HMT-160 | Named | 100. |
| 2 | cWee-6b1b | Bergeyella sp. HMT 322 | HMT-322 | Phylotype | 99.8 |
|  | Gran-5a37 | Granulicatella adiacens | HMT-534 | Named | 99.8 |
|  | Haem-7aaf | Haemophilus parainfluenzae | HMT-718 | Named | 100. |
|  | Pept-facb | Peptococcus sp. HMT 168 | HMT-168 | Phylotype | 99.5 |
|  | Porp-fd4f | Porphyromonas pasteri | HMT-279 | Named | 100. |
|  | Prev-fb41 | Prevotella aurantiaca | HMT-943 | Named | 99.8 |
|  | Prev-71e7 | Prevotella melaninogenica | HMT-469 | Named | 99.8 |
|  | Prev-04b7 | Prevotella nanceiensis | HMT-299 | Named | 99.8 |
|  | Prev-603e | Prevotella shahii | HMT-795 | Named | 100. |
|  | Stre-0531 | Streptococcus parasanguinis clade 411 | HMT-411 | Named | 99.5 |
|  | Veil-6e24 | Veillonella parvula | HMT-161 | Named | 99.5 |
